# Correction: Dassanayake et al. Synergistic Field Crop Pest Management Properties of Plant-Derived Essential Oils in Combination with Synthetic Pesticides and Bioactive Molecules: A Review. *Foods* 2021, *10*, 2016

**DOI:** 10.3390/foods13121874

**Published:** 2024-06-14

**Authors:** Mackingsley Kushan Dassanayake, Chien Hwa Chong, Teng-Jin Khoo, Adam Figiel, Antoni Szumny, Chee Ming Choo

**Affiliations:** 1 School of Pharmacy, Faculty of Science and Engineering, University of Nottingham Malaysia, Jalan Broga, Semenyih 43500, Malaysia; hyxmd1@nottingham.edu.my (M.K.D.); tengjin.khoo@nottingham.edu.my (T.-J.K.); 2 Department of Chemical and Environmental Engineering, Faculty of Science and Engineering, University of Nottingham, Jalan Broga, Semenyih 43500, Malaysia; 3 Institute of Agricultural Engineering, Wrocław University of Environmental and Life Sciences, Chełmónskiego 37a, 51-630 Wrocław, Poland; adam.figiel@upwr.edu.pl; 4 Department of Chemistry, Wrocław University of Environmental and Life Sciences, Norwida 25, 50-375 Wrocław, Poland; antoni.szumny@upwr.edu.pl; 5 Centre for Water Research, Faculty of Engineering, Built Environment and Information Technology, SEGi University Kota Damansara, Petaling Jaya 47810, Malaysia; choocheeming@segi.edu.my

## Missing Citation

In the original publication [1], reference [51] was not cited. The citation has now been inserted in Section 4. Pesticidal and Fungicidal Action Mechanisms of Plant-Derived Essential Oils. In Section 4.1. Mode of Action of Insecticidal Essential Oils, the second sentence should read “The EO metabolite-mediated inhibition of acetylcholinesterase (AChE) and octopamine pathways of insects [46–51] (Figure 1) has been well investigated and documented. Among these mechanisms, the inhibition of AChE is one of the most exploited, since AChE is an enzyme that plays a crucial role in neuromuscular and neuronal communication in insects [52–54].”

The citation has also been inserted in the caption of Figure 1 and should read “Figure 1. Insecticidal action mechanisms of plant-derived essential oils (e.g., [51]).”

## Add Reference

A new reference [51] was added in the reference part, and it appears below:

51. Jankowska, M.; Rogalska, J.; Wyszkowska, J.; Stankiewicz, M. Molecular Targets for Components of Essential Oils in the Insect Nervous System—A Review. *Molecules* **2018**, *23*, 34.

With this correction, the order of some references has been adjusted accordingly.

The authors state that the scientific conclusions are unaffected. This correction was approved by the Academic Editor. The original publication has also been updated.
